# Time Restricted Eating in Alzheimer’s Disease: TREAD

**DOI:** 10.1002/alz70861_108364

**Published:** 2025-12-23

**Authors:** Yonas E. Geda, DeJarra Rivera, Janina Krell‐Roesch, Isabella Zaniletti, Geetika Chahal, Tracy Smith, Candice DeCuna, Yehansa Hettiwatte, Emily Aliskevich, Jordan Gunning, Migbare Demeke, Nevine Khan, Danielle Eagan, Susan Racette

**Affiliations:** ^1^ Barrow Neurological Institute, Phoenix, AZ USA; ^2^ Karlsruhe Institute of Technology, Karlsruhe, Baden‐Württemberg Germany; ^3^ IZ Statistics LLC, Tampa, FL USA; ^4^ Arizona State University, Phoenix, AZ USA

## Abstract

**Background:**

Time Restricted Eating (TRE) may impact cognition favorably. TRE leads to a metabolic switch from glucose to fatty acid and ketone utilization, leading to a cascade of adaptive metabolic and cellular responses. Cortical glucose uptake is decreased in Alzheimer’s disease (AD), however, ketone uptake is preserved. Little is known about the impact of TRE in patients with mild cognitive impairment (MCI). We sought to investigate the feasibility of implementing a TRE intervention among patients with MCI.

**Method:**

Setting: Barrow Neurological Institute in Phoenix, USA. Design: Time Series design in which the participant serves as his/her own control with cognitive, behavioral and biomarker assessments at pre‐ and post‐interventions. Intervention: 16 hours of fasting and an 8 hour eating window daily, on approximately 5 Days/week, for 3 months. Meal tracking was monitored by an app. Intended sample : 30 study participants that meet the Mayo Clinic Criteria for MCI, age 60‐80 years old, BMI >18.5 and <40.0 kg/m^2^, and having a supportive study partner. Outcome measures for feasibility: participant recruitment and retention and metrics of acceptability, safety, and adherence to the intervention. Other outcomes: measures of attention and executive domains, behavioral measures, biomarkers (metabolic and blood based AD biomarkers) and exit surveys of a participant and partner.

**Result:**

To date we have recruited 22 participants: 15 men, 7 women. Median age: 71 years (range 63‐83). Preliminary results: participants followed the 16 hours fasting and 8 hours of eating window on 90% of the recommended 5 days/week throughout the 12‐week intervention. To date, 16 participants completed the intervention and pre‐and post‐intervention evaluations. About 80% of participants reported that they will incorporate TRE in their life style. 42% of study partners noticed positive changes.

**Conclusion:**

Preliminary observations from the TREAD trial indicate that a TRE intervention among patients with MCI is feasible and well‐tolerated.

